# Pathogen Profile of Children Hospitalised with Viral Respiratory Infections in Galati County, Romania

**DOI:** 10.3390/v17040586

**Published:** 2025-04-19

**Authors:** Elena-Roxana Matache (Vasilache), Gabriela Gurau, Cosmin-Raducu Raileanu, Anamaria Zaharescu, Gabriel Valeriu Popa, Nicoleta-Maricica Maftei, Camelia Busila, Madalina Nicoleta Matei, Dana Tutunaru

**Affiliations:** 1 Department of Morphological and Functional Sciences, Faculty of Medicine and Pharmacy, “Dunarea de Jos” University, 800008 Galati, Romania; elena.matache@ugal.ro; 2 Medical Laboratory Department, “Sf. Ioan” Emergency Clinical Hospital for Children, 800494 Galati, Romania; nicoleta.aron@ugal.ro; 3 Department of Dental Medicine, Faculty of Medicine and Pharmacy, “Dunarea de Jos” University, 800008 Galati, Romania; anamaria.zaharescu@ugal.ro (A.Z.); secretar.med@ugal.ro (G.V.P.); madalina.matei@ugal.ro (M.N.M.); 4 Department of Pharmaceutical Sciences, Faculty of Medicine and Pharmacy, “Dunarea de Jos” University, 80008 Galati, Romania; dana.tutunaru@ugal.ro; 5 “Sf. Ioan” Emergency Clinical Hospital for Children, 800494 Galati, Romania; camelia.busila@ugal.ro; 6 Medical Clinical Department, Faculty of Medicine and Pharmacy, “Dunarea de Jos” University, 800008 Galati, Romania; 7 Medical Laboratory Department, “Sf. Apostol Andrei” Emergency Clinical Hospital, 800578 Galati, Romania

**Keywords:** viral respiratory infections, RT-PCR, children

## Abstract

Respiratory infections are the most common infectious diseases among children, representing a cause of death and generating a significant number of hospitalisations. The aim of the study was to analyse the epidemiological and clinical characteristics of viral pathogens causing respiratory tract infections in newborns and young children admitted to Galati pediatric hospital between October 2022 and December 2023. The diagnosis was performed using multiplex RT-PCR panels, which allowed simultaneous identification of respiratory pathogens (viruses and bacteria). From a total of 803 hospitalised patients with respiratory diseases, 607 (75.6%) children had a positive result for at least one respiratory virus and 96 patients (11.9%) were identified with bacterial infections. Mixed coinfections were found in almost half of the patients (44.5%). Most of RSV positive children had an increased length of stay, more than 7 days. It was shown a decline in severe cases of viral respiratory infections with prolonged hospitalisation as patients age up to 5 years.

## 1. Introduction

Respiratory infections are a major public health problem among children, being the most common infectious diseases and having a significant impact on mortality and hospitalisations [1]. Globally, acute respiratory infections are responsible for approximately 20% of deaths in children under 5 years, with pneumonia being particularly dangerous for this age group [2], accounting for 15% of global deaths in 2017 [3]. In Romania, it has been recorded a higher rate of mortality in children under 5 years, reaching up to 25% [4].

Most acute respiratory tract infections (ARTIs) are caused by viral pathogens, such as respiratory syncytial virus (RSV), the leading cause of bronchiolitis in children under 2 years. Other important viruses responsible for ARTIs are rhinoviruses, parainfluenza viruses (PIVs), metapneumoviruses and influenza viruses (INFs). In addition, human coronaviruses, adenoviruses and bocaviruses have been identified less common but relevant pathogens [5,6].

Although detection rate of viral co-infections in hospitalised children has been reported from 10% to 30% [7], its clinical impact is not well known [8].

Moreover, there are other pathogens, including bacteria, fungi, parasites, Mycoplasma and Rickettsia [9], which play a key role in etiology of respiratory infections. The most common bacteria involved are *Mycoplasma pneumoniae*, *Staphylococcus aureus* and *Streptococcus pneumoniae*, which is considered to be a common pathogen that colonizes the nasopharynx of young children [5,6]. Less common bacteria have also been described in literature, like *Chlamydophila pneumoniae* or *Legionella pneumophila*, causing from mild to severe cases of respiratory infections [10]. Fungal infections, caused by *Aspergillus*, *Crytococcus* and *Pneumocystis*, remain localised in the lungs or disseminate to other tissues, affecting frequently immunocompromised patients [11].

To clinically diagnose respiratory infections is often difficult, due to similar symptoms produced by both viral and bacterial infections [12,13]. Diagnostic methods for viruses are limited, which often leads to unnecessary antibiotic administration, side effects and increased in antimicrobial resistance [14]. Therefore, rapid and accurate identification of pathogens causing viral infections is essential for early diagnosis and appropriate clinical decisions. The molecular method RT-PCR is considered the gold standard in diagnosing respiratory infections [15]. Simultaneous and accurate identification of multiple respiratory pathogens can be rapidly achieved by multiplex RT-PCR assays [16,17]. Thus, several primers are inserted into one reaction tube leading to amplification of genomic fragments of different pathogens [18].

The aim of this study was to give a broad overview on the epidemiological, etiological and clinical aspects of children hospitalised with acute respiratory tract infections.

## 2. Materials and Methods

### 2.1. Study Design and Configuration

This retrospective study was conducted at “Sf. Ioan” Children’s Emergency Hospital Galati, Romania, between October 2022 and December 2023, on a group of 803 pediatric patients.

The study was conducted with the approval of the Ethics Committee from “Sf. Ioan” Emergency Clinical Hospital for Children Galati, obtained retrospectively in accordance with the regulations applicable to retrospective research (No 29002 on 29 November 2024). Used data were previously collected within the framework of standard care procedures, without additional interventions on the patients. Therefore, specific informed consent was not required for this analysis.

### 2.2. Patient’s Enrolment

Inclusion criteria:

- hospitalised children of age 0–60 months
- acute fever (temperature ≥ 38 °C) or at least one respiratory symptom (cough, rhinorrhea, nasal congestion, wheezing or sore throat)
- undergoing the Allplex™ Respiratory Panel Assays kits 1,2,3,4
- onset of illness within 3 days before admission

Exclusion criteria:

- children admitted for other disorders

### 2.3. Sample Collection

Samples were collected individually from each child by trained healthcare personnel. Synthetic fiber swabs with plastic rods were used to collect nasopharyngeal exudates, which were placed in sterile tubes with 2–3 mL of viral transport medium (VTM) or universal transport medium (UTM). Samples were recorded, assigned a unique identification number, processed immediately or stored at −70 °C until PCR testing.

### 2.4. Extraction of Viral Nucleic Acids and Multiplex RT-PCR Analysis

Extraction of viral nucleic acids was performed with the Nimbus automated extractor (Seegene Inc., Seoul, Republic of Korea), using the STARMag 96 × 4 universal kit (Seegene Inc., Republic of Korea), according to the manufacturer’s instructions, 100 μL of nucleic acid were eluted from a volume of 300 μL sample. Multiplex RT-PCR panels were performed using the Allplex™ Respiratory Panel Assays kits 1,2,3,4 (Seegene Inc., Republic of Korea) for the identification of 12 viruses and 7 respiratory bacteria: SARS-CoV-2, Influenza A/B, RSV, human adenovirus, human metapneumovirus, human enterovirus, human parainfluenza 1/2/3/4, human bocavirus 1/2/3/4, human coronaviruses (229E, NL63, OC43), human rhinovirus A/B/C, *Chlamydophila pneumoniae*, *Mycoplasma pneumoniae*, *Legionella pneumophila*, *Bordetella pertussis*, *Bordetella parapertussis*, *Streptococcus pneumoniae* and *Haemophilus influenzae*. qRT-PCR was performed with the CFX96 amplifier (BIO-RAD, USA). All RT-PCR assays were validated with an internal control.

We used Seegene Viewer software version 3, in which the threshold Cycle (Ct) were automatically determined. The positive result was set at amplification of viral and bacterial targeted genes within the cut-off values < 42. However, we considered the result positive at Ct value < 39.

Analytical sensitivity and specificity of Allplex tests consisted of a lower limit of detection equal to the lowest concentration measured in 95% of the replicates.

### 2.5. Statistical Analysis

We performed statistical analyses using IBM SPSS Statistics version 26 software with a significance level up to 0.05. Categorical variables were expressed as counts and percentages, while continuous variables were presented as means ± standard deviation. Kolmogorov-Smirnov test was used to check the normality of the data. Continuous variables were analysed with Mann-Whitney U test as variables were not normally distributed. Categorical variables were analysed using χ^2^ test or Fisher’s exact test.

## 3. Results

Out of a total of 803 patients, 57% (*n* = 458) were boys and 43% (*n* = 345) were girls.

Viral monoinfections were observed in 195 patients (24.3%), 45 children (5.6%) had coinfections with 2 viruses and only 10 patients (1.2%) had coinfections with 3 viruses. Viral-bacterial coinfections were found in 357 patients (44.5%). Bacterial monoinfections represented 9.1% (*n* = 73), while bacterial coinfections were only 2.9% (*n* = 23). No pathogens were found in 12.5% (*n* = 100).

Viral respiratory infections identified with at least one virus were found in 75.6% (*n* = 607) of children, of which 57.5% (*n* = 349) were boys and 42.5% (*n* = 258) were girls.

**Figure 1:**
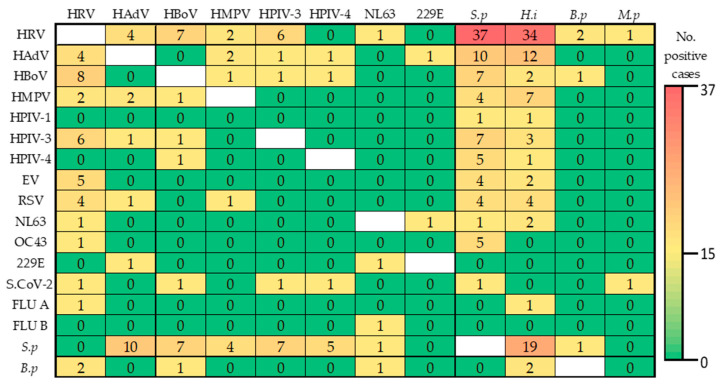
Heat map showing coinfection of two respiratory pathogens. HRV: Human rhinovirus; HAdV: Human adenovirus; HBoV: Human bocavirus; HMPV: Human metapneumovirus; HPIV: Human parainfluenza virus; EV: Enterovirus; RSV: Respiratory syncytial virus; HCoV (NL63, OC43, 229E): Human coronaviruses; FLU A/B: Influenza A/B; *S.p*: *S. pneumoniae*; *H.i*: *H. influenzae*; *B.p*: *B. parapertussis*; *M.p*: *M. pneumoniae*.

The most common viral-bacterial coinfections identified were HRV + *S. pneumoniae* (*n* = 37, 6.1%), HRV + *H. influenzae* (*n* = 34, 5.6%) and HRV + *S. pneumoniae* + *H. influenzae* (*n* = 33, 5.4%), while HRV + HBoV 1/2/3/4 (*n* = 7, 1.2%) represented the most frequent viral coinfections diagnosed (Figure 1 and Table A1).

**Figure 2:**
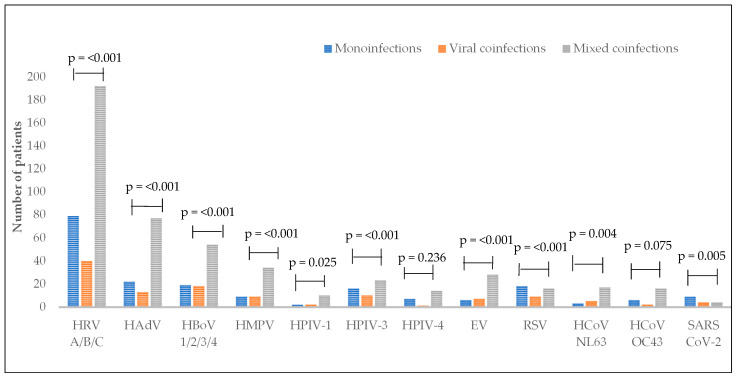
Comparison of frequency of pathogens detected in monoinfections and coinfections.

We showed the frequency of pathogens according to mono- and coinfections in Figure 2. SARS-CoV-2 was observed mostly in monoinfections (*p* = 0.008), while HRV A/B/C, HAdV, HBoV 1/2/3/4, HMPV, HPIV-3, EV and HCoV NL63 were increased in mixed coinfections, with significant *p* values of <0.001, <0.001, <0.001, <0.001, <0.001, <0.001 and 0.004, respectively. By contrast, RSV was found less in viral coinfections, than in mono- and mixed coinfections (*p* ≤ 0.001).

No significant statistical difference was observed when viral etiology and distribution of monoinfections and coinfections were evaluated according to gender. The most frequent pathogens found in boys and girls were: HRV A/B/C (38.9%, 38%), HAdV (13.1%, 14.5%,) and HBoV 1/2/3/4 (10.5%, 12.5%) (Table A2).

Most respiratory infections were found in patients up to 12 months (*n* = 248, 35%), followed by those aged between 13 and 24 months (*n* = 140, 20%), 25- 36 months (*n* = 114, 16%), 37–48 months (*n* = 117, 17%) and 49–60 months (*n* = 84, 12%). HRV was the most frequent pathogen involved in all age groups, mainly affecting children aged 2–5 years. (*p* = 0.010). RSV (9.5%) was a common factor in the youngest group (0–12 months) (*p* = 0.001), while HboV 1/2/3/4 (19.7%) and HPIV-3 (10.4%) had the highest rate in the age group 13–24 months (*p* ≤ 0.001, respectively 0.017). HadV (20%) was the most frequently detected virus among children 37–48 months of age (*p* = 0.004). Regarding the number of pathogens involved in respiratory infection, viral monoinfections were increased in newborns and infants up to 12 months (29.5%), while viral coinfections were more common in children aged 37–48 months (11.5%). Bacterial monoinfections and mixed coinfections prevailed in patients aged 25–36 months (11.5%, respectively 52.5%). The oldest age group had a higher rate of bacterial coinfections (4.3%) (*p* ≤ 0.001) (Table 1).

HRV A/B/C has been circulating throughout the year, with a higher rate recorded in fall season (50.6%). HadV (23.4%) and HMPV (14.9%) were detected mainly in spring, while HPIV-3 (20.4%) and EV (9.7%) infections increased during summer. RSV (12.8%) had its peak in winter. We found no significant difference between seasons and rate of mono- or coinfections. (Table 2).

Based on the mean length of stay of our study participants (7 days), we divided them into 2 categories: short or normal length of stay for children admitted between 1–7 days, and prolonged hospitalisation for patients who exceeded 7 days. We observed that children up to 1 year were associated with a prolonged length of stay (*p* = 0.001). Amongst all viral agents evaluated, only RSV had a significant result in terms of prolonged hospitalisation (*p* ≤ 0.001). Apart from that, no statistical difference was observed between gender, number of pathogens and length of stay (Table 3).

Clinical features and treatment are provided in Table 4. Lower respiratory tract infections (LRTI) were the most common (84.4%, *n* = 678), such as pneumonia 79.1% (*n* = 635), bronchitis 3.7% (*n* = 30) and bronchiolitis 1.6% (*n* = 13). Pneumonia predominated in all types of infections, especially in bacterial monoinfections (84.9%), whereas bronchiolitis was detected mainly in viral monoinfections (3.1%). (*p* ≤ 0.001).

A small proportion of cases (15.6%, *n* = 125) was diagnosed with upper respiratory tract infections (URTI), most of which were rhinopharyngitis (4.5%, *n* = 36), pharyngitis (3.2%, *n* = 26), tonsillitis (2.2%, *n* = 18), pharyngotonsillitis (1.9%, *n* = 15) and laryngitis (0.4%, *n* = 3). Viral coinfections with 2 viruses were frequently found in both upper (84.4%) and lower (84.4%) respiratory tract infections.

Among all respiratory viruses analised, SARS-CoV-2 and HadV were reported more in URTI (*p* = 0.004, respectively *p* = 0.014) while RSV, HRV A/B/C and HBoV 1/2/3/4 were associated with LRTI (*p* = 0.008, *p* = 0.014, respectively *p* = 0.045) (Table A3).

Cough was one of the main respiratory symptoms, which predominated in both mono- and coinfections, followed by wheezing that was associated with viral monoinfections (11.3%) (*p* = 0.029). A small number of patients developed sepsis (3.9%, *n* = 31), most cases (*n* = 10) were found in children with negative PCR results (*p* = 0.045). Moreover, almost half of SARS-CoV-2 positive (47.2%) required PICU admission (*p* = 0.017) (Table A4). Regarding the treatment, antibiotics were prescribed in 94% of cases (*n* = 755).

## 4. Discussion

In this retrospective study, we evaluated the prevalence, viral profile, and clinical characteristics of respiratory tract infections in children under 5 years from a county in southeast of Romania. The importance of this study is given by the limited epidemiological data available in literature on multiple viral pathogens responsible for respiratory infections, affecting pediatric population of Romania. The study was conducted by the end of 6th wave of SARS-CoV-2 infections, starting from autumn 2022 to December 2023.

The viral detection rate was 75.6%, while in literature different results were reported: 90.1% in Senegal [19], 89.2% in Turkey [20], 85.5% in Portugal [8], 83.8% in Ethiopia [21], 82.5% in Bangladesh [6], and 78.1% in Bulgaria [22]. Lower rates of confirmed viral infections were detected in Czech Republic 52% [23] and 64% in Poland and Saudi Arabia [24,25].

Among the patients enrolled in the study with viral respiratory tract infections, 24.3% had monoinfections, 5.6% had coinfections with 2 viruses and only 1.2% had viral coinfections with 3 pathogens. In literature, monoinfections ranged from 54.5% to 89.3% [6,8,12,17,20,23,25,26]. Neli Korsun et al. [22] and Gulsen Akkoc et al. [20] reported a higher detection of viral coinfections with 2 agents–11.3% and 23.6%, respectively, while the rate of coinfections with 3 viral pathogens ranged less from 1.2% to 4.6% [20,22].

A study conducted in Barcelona [27] revealed that HboV was more frequently identified in the age group 12–24 months (55.03%) and patients aged 2–4 years were more exposed to EV (47.55%), HadV (43.23%) and HRV (34.4%). These findings are similar to our study. We found a lower detection rate of HBoV positive cases (11.3%), but also more prevalent in age group 13–24 months. Depending on the country, HBoV detection rates ranged from 38% to 84% according to data published in the review by Trapani S et al. [28]. It’s worth mentioning that prior research highlighted HBoV as being among the most virulent respiratory tract viruses [29].

Previous studies from Spain, Morocco, Taiwan and Germany [27,30,31,32] have shown a predominance of RSV in children under 12 months, in contrast to our study where detection rate was low (9.5%). We found HRV to be the most frequently involved in this age group, with a percentage of 31.5%. Similarly, in a study conducted in Madrid, Cristina Calvo et al. [33] reported an increased frequency of HRV in children under one year, with a much higher rate of 71%. Also, Nikolaos J. Tsagarakis et al. [34], in a study from Greece, observed HRV virus predominant in children under 2 years. Jean Richter et al. [35] showed in their study conducted in Cyprus that HadV was more frequently found in children up to 3 years (11%), different from the result in our study where we noticed a significant increase with age, from 7.9% in children up to one year, reaching a detection rate of 20% in children between 37–48 months (Table 1). In another study from Northeast of Italy [36] it has been found a higher distribution of SARS-CoV-2 infections in children under 2 years of age (8.4%) and a higher detection rate with positive HBoV in children between 2–5 years of age (6.5%).

We analysed the frequency of pathogens in mono- and coinfections and we found SARS-CoV-2 prevailing in monoinfections (9/17, 52.9%), result which is consistent with previous studies [37,38]. HRV A/B/C, HadV, HBoV 1/2/3/4 and HMPV were found more in mixed coinfections (Figure 2). The most frequent combinations in viral coinfections were HRV A/B/C/HBoV (*n* = 7) and HRV A/B/C/HPIV-3 (*n* = 6). Similar findings were reported by Cedric Mantelli et al. in Southern France [26]. However, different studies showed HRV/AdV, HRV/HCoV-OC43, and HRV/FluA being mostly detected [7].

The impact of viral coinfections on children represents a topic of interest in literature. Previous studies have shown that HRV + non-RSV combinations have a short hospital stay [39], while in a recent analysis of 63 patients hospitalised in PICU, M.Duyu et al. showed that HRV + non-RSV coinfection is associated with severe evolution. [29]. In a cohort study of 164 children under 3 years, Guocui Zhang et al. [40] found higher rates of viral coinfections in children aged 13–24 months. We observed viral coinfections increasing in children aged 37–48 months (11.5%), followed by 13–24 months group (7.2%), a slightly different result due to study inclusion criteria of children up to 5 years (Table 1).

It is unknown whether coinfections increase or reduce disease severity [37]. According to literature data, viral coinfections can follow 2 pathways consisting in viral accommodation which leads to coexistence of viruses or survival of a single virus by inhibiting the other [41]. Rhinovirus, known as a fast replicating virus, can inhibit the replication of other viruses. Moreover, it has been suggested that coinfections with HRV have lower risk of complications [42]. By contrast, HPIV is a slow replicator and its replication can be inhibited by other viruses [43].

In the current study, bacterial coinfections were identified in more than a third of children (44.5%). Previous studies have reported lower detection rates ranging from 16% to 37% [29,44]. The most common combinations of viral-bacterial coinfections identified were HRV + *S. pneumoniae* (*n* = 37, 6.1%), HRV + *H. influenzae* (*n* = 34, 5.6%) and HRV + *S. pneumoniae* + *H. influenzae* (*n* = 33, 5.4%). Nevine R. El Baroudy et al., also reported HRV + *S. pneumoniae* as the most frequent viral-bacterial combination found (*n* = 6) [44]. It has been shown that viral-bacterial infections can increase viral susceptibility. For instance, *S. pneumoniae*, is a predisposing factor in children under 2 years to HMPV infections [45], while *H. influenzae* facilitates the entry of HRV by stimulating adhesion proteins found in epithelial cells [46].

Furthermore, we have observed a decline in severe cases of viral respiratory infections with prolonged hospitalisation as patients age up to 5 years, as it has been found in literature [47]. In a recent study, Abdoul Kader Ilboudo et al. reported an association between HcoV OC43 and Influenza B with prolonged length of stay in children under 5 years [48]. We found no association between them, possibly due to low number of positive cases. Our results showed that RSV caused a higher number of prolonged hospitalisations, in line with other studies [27,49,50]. Interestingly, almost half the patients tested positive for SARS-CoV-2 required PICU admission, contradicting other studies which reported milder cases and low rate of PICU admission (4.5%) [51]. Nevertheless, many different studies reported SARS-CoV-2 being a cause of death in children [52,53].

Regarding etiology of respiratory infections, Chun-Yu Yen et al. found that FLU B and HCoV viruses were associated with URTI in children [54]. In another study, Fiseha Wadilo et al. reported FLU A being corelated with URTI [21]. By contrast, we observed a significant association of SARS-CoV-2 and HAdV with URTI. As for LRTI, it has been shown that RSV is the most common cause [55,56], followed by FLU, PIV and HAdV [55]. Our results revealed, besides RSV, different viruses being involved in LRTI, such as HRV and HBoV.

It is essential to differentiate viral from bacterial respiratory tract infection, in order to avoid unnecessary antibiotic usage. Although viral etiology has been identified, the rate of antibiotics in our study is high (94%). A possible explanation could be the fact that viral pathogens can lead to severe infections [57]. Moreover, antibiotic therapy is known to lower the risk of death from respiratory infections, but their inappropriate use can develop antimicrobial resistance [58].

Viral respiratory infections can be detected regardless of the season, with peaks during cold weather. However, variations can be found in different regions due to climate factors, social diversity or medical infrastructure. Suling Li et al. revealed in a research conducted in China that HRV was widespread during all seasons, but winter [59]. In our analyses, HRV has been circulating throughout the year, similar to other studies from Ukraine and Morocco [30,60], highest rate being recorded in fall and least during summer. A viable explanation could be that HRV, considered a non-enveloped virus, is characterized by resistance to ethanol-based disinfectants [61] and long survival on environmental surfaces [62]. We found HBoV more prevalent in fall season, while HAdV and HMPV were detected mainly in spring, as previous studies reported [22,27,30]. In another study from Ukraine, it has been reported HAdV circulating more during winter and HMPV having higher prevalence in fall-winter [60]. Additionally, our research confirmed that RSV peaked in winter [25,27,30,32,63] and EV was more prevalent during summer [39].

The study has several limitations, one of which is that we have only analised hospitalized patients, so children with mild symptoms were underrepresented. Another important limitation worth mentioning is the lack of certain data, like immunization against some pathogens or antibiotic treatment taken before hospitalisation. Moreover, the results of our study were obtained from a single hospital in one region, which cannot be representative of the entire pediatric population in our country.

## 5. Conclusions

A high prevalence of acute respiratory tract infections was found amongst hospitalised children in southeast of Romania. Understanding viral epidemiological trend and clinical course is important not only for preventive strategies, but also for an adequate management. Further studies are needed to evaluate the severity of coinfections.

## Figures and Tables

**Table 1:** Distribution of respiratory pathogens by age groups.

Viral Pathogen	0–12 Months (%) *n* = 305	13–24 Months (%) *n* = 152	25–36 Months (%) *n* = 122	37–48 Months (%) *n* = 130	49–60 Months (%) *n* = 94	Total (%) *n* = 803	*p* Value
HRV A/B/C	96 (31.5)	58 (38.2)	52 (42.6)	63 (48.5)	40 (42.6)	309 (38.5)	0.010 *
HAdV	24 (7.9)	24 (15.8)	19 (15.6)	26 (20)	17 (18.1)	110 (13.7)	0.004 *
HBoV 1/2/3/4	22 (7.2)	30 (19.7)	22 (18)	14 (10.8)	3 (3.2)	91 (11.3)	<0.001 *
HMPV	26 (8.5)	7 (4.6)	7 (5.7)	8 (6.2)	4 (4.3)	52 (6.5)	0.421
HPIV-1	3 (1)	1 (0.7)	3 (2.5)	4 (3.1)	2 (2.1)	13 (1.6)	0.331
HPIV-2	2 (0.7)	1 (0.7)	1 (0.8)	0 (0)	0 (0)	4 (0.5)	0.947
HPIV-3	22 (7.2)	16 (10.4)	2 (1.6)	6 (4.6)	3 (3.2)	49 (6.1)	0.017 *
HPIV-4	9 (3)	2 (1.3)	3 (2.5)	5 (3.9)	3 (3.2)	22 (2.7)	0.752
EV	13 (4.3)	4 (2.6)	12 (9.8)	6 (4.6)	4 (4.3)	39 (4.9)	0.075
RSV	29 (9.5)	6 (4)	5 (4.1)	1 (0.8)	2 (2.1)	43 (5.4)	0.001 *
HCoV NL63	11 (3.6)	5 (3.3)	3 (2.5)	2 (1.5)	3 (3.2)	24 (3)	0.853
HCoV OC43	12 (3.9)	6 (4)	2 (1.6)	3 (2.3)	1 (1.1)	24 (3)	0.548
HCoV 229E	0 (0)	0 (0)	1 (0.8)	0 (0)	1 (1.1)	2 (0.4)	0.098
SARS-CoV-2	9 (3)	3 (2)	1 (0.8)	3 (2.3)	1 (1.1)	17 (2.1)	0.739
FLUA	2 (0.7)	0 (0)	1 (0.8)	3 (2.3)	1 (1)	7 (0.9)	0.257
FLU B	2 (0.7)	0 (0)	0 (0)	0 (0)	0 (0)	2 (0.3)	1.000
Number of pathogens Viral monoinfections	90 (29.5)	31 (20.4)	27 (22.1)	25 (19.2)	22 (23.4)	195 (24.3)	<0.001 *
Viral coinfections	18 (5.9)	11 (7.2)	7 (5.7)	15 (11.5)	4 (4.3)	55 (6.9)	
Mixed coinfections	109 (35.7)	77 (50.7)	64 (52.5)	63 (48.5)	44 (46.8)	357 (44.5)	
Bacterial monoinfections	23 (7.5)	17 (11.2)	14 (11.5)	9 (6.9)	10 (10.6)	73 (9.1)	
Bacterial coinfections	8 (2.6)	4 (2.6)	2 (1.6)	5 (3.9)	4 (4.3)	23 (2.9)	
Total	248 (81.3)	140 (92.1)	114 (93.4)	117 (90)	84 (89.4)	703 (87.6)	

* *p* < 0.05. HRV: Human rhinovirus; HAdV: Human adenovirus; HBoV: Human bocavirus; HMPV: Human metapneu-movirus; HPIV: Human parainfluenza virus; EV: Enterovirus; RSV: Respiratory syncytial virus; HCoV: Human coronaviruses; FLU A/B: Influenza A/B.

**Table 2:** Distribution of respiratory pathogens by season.

Pathogens	Spring *n*, % *n* = 175	Summer *n*, % *n* = 93	Autumn *n*, % *n* = 269	Winter *n*, % *n* = 266	*p* Value
Viral monoinfections	33 (18.9)	19 (20.4)	80 (29.7)	63 (23.7)	0.055
Viral coinfections	13 (7.4)	3 (3.2)	20 (7.4)	19 (7.1)	
Mixed coinfections	95 (54.3)	42 (45.2)	116 (43.1)	104 (39.1)	
Bacterial monoinfections	12 (6.9)	11 (11.8)	23 (8.6)	27 (10.2)	
Bacterial coinfections	2 (1.1)	4 (4.3)	5 (1.9)	12 (4.5)	
Viral pathogens HRV A/B/C	70 (40)	27 (29)	136 (50.6)	76 (28.6)	<0.001 *
HAdV	41 (23.4)	14 (15.1)	20 (7.4)	35 (13.2)	<0.001 *
HBoV 1/2/3/4	11 (6.3)	5 (5.4)	42 (15.6)	33 (12.4)	0.005 *
HMPV	26 (14.9)	1 (1.1)	2 (0.7)	23 (8.7)	<0.001 *
HPIV-1	4 (2.3)	0 (0)	8 (3)	1 (0.4)	0.045 *
HPIV-2	3 (1.7)	0 (0)	1 (0.4)	0 (0)	0.109
HPIV-3	8 (4.6)	19 (20.4)	18 (6.7)	4 (1.5)	<0.001 *
HPIV-4	3 (1.7)	3 (3.2)	12 (4.5)	4 (1.5)	0.161
EV	5 (2.9)	9 (9.7)	21 (7.8)	4 (1.5)	0.001 *
RSV	0 (0)	0 (0)	9 (3.4)	34 (12.8)	<0.001 *
HCoV NL63	17 (9.7)	2 (2.2)	0 (0)	5 (1.9)	<0.001 *
HCoV OC43	1 (0.6)	0 (0)	7 (2.6)	16 (6)	0.002 *
HCoV 229E	1 (0.6)	0 (0)	0 (0)	1 (0.4)	0.632
SARS-CoV-2	4 (2.3)	2 (2.2)	8 (3)	3 (1.1)	0.515
FLUA	1 (0.6)	0 (0)	0 (0)	6 (2.3)	0.028 *
FLUB	1 (0.6)	0 (0)	0 (0)	1 (0.4)	0.632

* *p* < 0.05. HRV: Human rhinovirus; HAdV: Human adenovirus; HBoV: Human bocavirus; HMPV: Human meta-pneumovirus; HPIV: Human parainfluenza virus; EV: Enterovirus; RSV: Respiratory syncytial virus; HCoV: Hu-man coronaviruses; FLU A/B: Influenza A/B.

**Table 3:** Factor analysis according to prolonged length of stay (LOS).

Variables	Positive *n* (%) *n* = 703	Prolonged LOS (>7 Days) *n* (%) *n* = 230	*p* Value
Gender Male Female	404 (57.5) 299 (42.5)	133 (57.8) 97 (42.2)	0.893
Age groups 0–12 months 13–24 months 25–36 months 37–48 months 49–60 months	248 (35.3) 140 (19.9) 114 (16.2) 117 (16.6) 84 (12)	106 (46.1) 37 (16.1) 29 (12.6) 30 (13) 28 (12.2)	0.001 *
Type of infections Monoviral Two viruses Three viruses Monobacterial Bacterial coinfection Mixed coinfection	195 (27.7) 45 (6.4) 10 (1.4) 73 (10.4) 23 (3.3) 357 (50.8)	72 (31.3) 14 (6.1) 1 (0.4) 16 (7) 8 (3.5) 119 (51.7)	0.160
HRV A/B/C	309 (44)	100 (43.5)	0.716
HAdV	110 (15.7)	29 (12.6)	0.105
HBoV 1/2/3/4	91 (12.9)	37 (16.1)	0.105
HMPV	52 (7.4)	19 (8.3)	0.589
HPIV-1	13 (1.9)	4 (1.7)	1.000
HPIV-2	4 (0.6)	1 (0.4)	1.000
HPIV-3	49 (7)	18 (7.8)	0.580
HPIV-4	22 (3.1)	11 (4.8)	0.108
EV	39 (5.6)	10 (4.4)	0.309
RSV	43 (6.1)	25 (10.9)	<0.001 *
HCoV NL63	24 (3.4)	7 (3)	0.827
HCoV OC43	24 (3.4)	8 (3.5)	1.000
HCoV 229E	3 (0.4)	0 (0)	-
SARS-CoV-2	17 (2.4)	4 (1.7)	0.603
FLU A	7 (1)	2 (0.9)	1.000
FLU B	2 (0.3)	1 (0.4)	0.553

* *p* < 0.05. HRV: Human rhinovirus; HAdV: Human adenovirus; HBoV: Human bocavirus; HMPV: Human metapneumovirus; HPIV: Human parainfluenza virus; EV: Enterovirus; RSV: Respiratory syncytial virus; HCoV: Human coronaviruses; FLU A/B: Influenza A/B.

**Table 4:** Comparison of clinical and management data between different types of respiratory infections.

Variables	Viral Mono-Infection *n* (%) *n* = 195	Coinfection 2 Viruses *n* (%) *n* = 45	Coinfection 3 Viruses *n* (%) *n* = 10	Bacterial Mono- Infection *n* = 73	Bacterial Coinfection *n* (%) *n* = 23	Mixed co- Infections *n* (%) *n* = 357	Negative PCR Result *n* (%) *n* = 100	*p* Value
Fever	102 (52.3)	26 (57.8)	4 (40)	44 (60.3)	9 (39.1)	208 (58.3)	51 (51)	0.326
Cough	142 (72.8)	30 (66.7)	10 (100)	57 (78.1)	20 (87)	290 (81.2)	63 (63)	0.001 *
Wheezing	22 (11.3)	1 (2.2)	0	1 (1.4)	2 (8.7)	25 (7)	3 (3)	0.029 *
Rhinorrhea	30 (15.4)	7 (15.6)	1 (10)	14 (19.2)	2 (8.7)	81 (22.7)	16 (16)	0.245
Dyspnoea	62 (31.8)	12 (26.7)	3 (30)	12 (16.4)	6 (26.1)	117 (32.8)	23 (23)	0.099
Nasal obstruction	20 (10.3)	2 (4.4)	2 (20)	9 (12.3)	2 (8.7)	32 (9)	11 (11)	0.636
Upper RTIs Lower RTIs	31 (15.9) 163 (83.6)	5 (11.1) 40 (88.9)	2 (20) 8 (80)	6 (8.2) 67 (91.8)	3 (13) 20 (87)	38 (10.6) 319 (89.4)	23 (23) 77 (77)	0.034 *
SpO_2_ < 92%	8 (4.1)	4 (8.9)	0	3 (4.1)	1 (4.4)	24 (6.7)	6 (6)	0.666
Sepsis	4 (2.1)	3 (6.7)	0	2 (2.7)	1 (4.4)	11 (3.1)	10 (10)	0.045 *
PICU admission	37 (19)	8 (17.8)	2 (20)	12 (16.4)	5 (21.7)	81 (22.7)	24 (24)	0.828
Death	1 (0.5)	0	0	0	0	1 (0.3)	4 (4)	0.066
Chest X-ray Interstitial Opacity Condensation Normal	123 (63.1) 14 (7.2) 1 (0.5) 6 (3.1)	27 (60) 2 (4.4) 0 1 (2.2)	7 (70) 0 0 1 (10)	51 (69.9) 10 (13.7) 1 (1.4) 2 (2.7)	15 (65.2) 3 (13) 0 1 (4.4)	222 (62.2) 36 (10.1) 3 (0.8) 12 (3.4)	63 (63) 7 (7) 1 (1) 2 (2)	0.073
Diagnosis at discharge Lower RTIs Pneumonia Bronchitis Bronchiolitis Upper RTIs (pharyngitis, laryngitis etc.)	150 (76.9) 6 (3.1) 6 (3.1) 32 (16.4)	38 (84.4) 1 (2.2) 1 (2.2) 38 (84.4)	8 (80) 0 0 2 (20)	62 (84.9) 3 (4.1) 2 (2.7) 8 (11)	19 (82.6) 1 (4.4) 0 3 (13)	290 (81.2) 1 (0.3) 3 (0.8) 38 (10.6)	68 (68) 3 (3) 1 (1) 23 (23)	<0.001 *
Oxygen therapy	10 (5.1)	2 (4.4)	0	1 (1.4)	1 (4.4)	12 (3.4)	5 (5)	0.746
Antibiotics	182 (93.3)	43 (95.6)	8 (80)	70 (95.9)	20 (87)	342 (95.8)	90 (90)	0.066

* *p* < 0.05. HRV: Human rhinovirus; HAdV: Human adenovirus; HBoV: Human bocavirus; HMPV: Human metapneumovirus; HPIV: Human parainfluenza virus; EV: Enterovirus; RSV: Respiratory syncytial virus; HCoV: Human coronaviruses; FLU A/B: Influenza A/B; PICU: pediatric intensive care unit; RTIs: respiratory tract infections.

## Data Availability

The raw data supporting the conclusions of this article will be made available by the authors on request.

## Appendix A

**Table A1:** Codetection of ≥3 respiratory pathogens.

Pathogens	*n* (%)
HRV A/B/C + *S. pneumoniae* + *H. influenzae*	33 (5.4)
HAdV + HRV A/B/C + *H. influenzae*	11 (1.8)
HMPV + *S. pneumoniae* + *H. influenzae*	10 (1.7)
HRV A/B/C + HBoV 1/2/3/4 + *S. pneumoniae* + *H. influenzae* HBoV 1/2/3/4 + HRV A/B/C + *S. pneumoniae*	9 (1.5) 6 (1)
HAdV + HRV A/B/C + *S. pneumoniae* + *H. influenzae*	6 (1)
HAdV + *S. pneumoniae* + *H. influenzae*	5 (0.8)
HAdV + HRV A/B/C + *S. pneumoniae*	5 (0.8)
HBoV 1/2/3/4 + HRV A/B/C + *H. influenzae*	5 (0.8)
EV + HRV A/B/C + *S. pneumoniae* + *H. influenzae*	5 (0.8)
EV + HRV A/B/C + *S. pneumoniae* HBoV 1/2/3/4 + *S. pneumoniae* + *H. influenzae*	5 (0.8) 4 (0.7)
RSV + *S. pneumonie* + *H. influenzae*	3 (0.5)
HPIV-4 + *S. pneumoniae* + *H. influenzae*	3 (0.5)
HCoV OC43 + *S. pneumoniae* + *H. influenzae*	3 (0.5)
EV + *S. pneumoniae* + *H. influenzae*	3 (0.5)
HPIV-3 + *S. pneumoniae* + *H. influenzae*	3 (0.5)
HPIV-3 + HRV A/B/C + *H. influenzae*	2 (0.3)
HPIV-3 + HRV A/B/C+ *S. pneumoniae*	2 (0.3)
HPIV-3 + HRV A/B/C + *S. pneumoniae* + *H. influenzae*	2 (0.3)
RSV + HRV A/B/C + *S. pneumoniae* + *H. influenzae*	2 (0.3)
HAdV + HMPV + *S. pneumoniae*	2 (0.3)
HAdV + HBoV 1/2/3/4 + *H. influenzae*	2 (0.3)
HAdV + HBoV 1/2/3/4 + *S. pneumoniae* + *H. influenzae*	2 (0.3)
HCoV NL63 + HBoV 1/2/3/4 + *S. pneumonie*	2 (0.3)
HCoV NL63 + HAdV + *S. pneumoniae* + *H. influenzae* HCoV OC43 + HRV A/B/C + *S. pneumoniae*	2 (0.3) 1 (0.2)
RSV + HAdV + *H. influenzae*	1 (0.2)
RSV + HAdV + HBoV 1/2/3/4	1 (0.2)
RSV + HCoV OC43 + *S. pneumoniae* + *H. influenzae*	1 (0.2)
RSV + HAdV + HRV A/B/C	1 (0.2)
RSV + HAdV + HRV A/B/C + *S. pneumoniae* + *H. influenzae*	1 (0.2)
RSV + HBoV 1/2/3/4 + HCoV OC43	1 (0.2)
FLU A + HAdV + *H. influenzae*	1 (0.2)
FLU A + *S. pneumoniae* + *H. influenzae*	1 (0.2)
FLU A + HBoV 1/2/3/4 + *S. pneumoniae* + *H. influenzae*	1 (0.2)
SARS-CoV-2 + HAdV + *S. pneumoniae*	1 (0.2)
SARS-CoV-2 + HRV A/B/C + *S. pneumoniae* + *H. influenzae*	1 (0.2)
SARS-CoV-2 + HBoV 1/2/3/4 + HRV A/B/C	1 (0.2)
HPIV-1 + EV + *S. pneumoniae* HPIV-1 + HAdV + HMPV + *H. influenzae*	1 (0.2) 1 (0.2)
HPIV-1 + HBoV 1/2/3/4 + *S. pneumoniae*	1 (0.2)
HPIV-1 + *S. pneumoniae* + *H. influenzae* HPIV-1 + HAdV + HMPV HPIV-1 + *B.parapertussis* + *S. pneumoniae* + *H. influenzae*	1 (0.2) 1 (0.2) 1 (0.2)
HAdV + HRV A/B/C + HPIV-3 + *S. pneumoniae*	1 (0.2)
HAdV + EV + *H. influenzae* HAdV + EV + HRV A/B/C	1 (0.2) 1 (0.2)
HAdV + HRV A/B/C + HBoV 1/2/3/4 + *H. influenzae* HAdV + *B.parapertussis* + *S. pneumoniae* + *H. influenzae* HAdV + HCoV OC43 + *S. pneumoniae* + *H. influenzae* HAdV + *S. pneumoniae* + *M. pneumoniae* + *H. influenzae* HAdV + HRV A/B/C + *S. pneumoniae* + *H. influenzae* + *M. pneumoniae* HAdV + HRV A/B/C + HCoV NL63 + *S. pneumoniae* + *H. influenzae* HAdV + HRV A/B/C + HBoV 1/2/3/4 + *S. pneumoniae* + *H. influenzae*	1 (0.2) 1 (0.2) 1 (0.2) 1 (0.2) 1 (0.2) 1 (0.2) 1 (0.2)
HCoV OC43 + HBoV 1/2/3/4 + *S. pneumoniae* HCoV OC43 + HRV A/B/C + *H. influenzae* HCoV OC43 + HRV A/B/C + *S. pneumoniae* + *H. influenzae*	1 (0.2) 1 (0.2) 1 (0.2)
HBoV 1/2/3/4 + *B.parapertussis* + *S. pneumoniae* HBoV 1/2/3/4 + HRV A/B/C + HCoV NL63 + *S. pneumoniae* HBoV 1/2/3/4 + HRV A/B/C + HCoV NL63	1 (0.2) 1 (0.2) 1 (0.2)
HRV A/B/C + HMPV + *H. influenzae*	1 (0.2)
HRV A/B/C + HBoV 1/2/3/4 + HMPV	1 (0.2)
HRV A/B/C + *B.parapertussis* + *H. influenzae*	1 (0.2)
HRV A/B/C + HCoV 229E + HCoV NL63 HRV A/B/C + *M. pneumoniae* + *H. influenzae* HRV A/B/C + *S. pneumoniae* + *H. influenzae* + *M. pneumoniae*	1 (0.2) 1 (0.2) 1 (0.2)
HMPV + HRV A/B/C + HCoV NL63 + *S. pneumoniae*	1 (0.2)
HMPV + HBoV 1/2/3/4 + *H. influenzae*	1 (0.2)
HMPV + HBoV 1/2/3/4 + *S. pneumoniae* + *H. influenzae*	1 (0.2)
HMPV + HAdV + *S. pneumoniae* + *H. influenzae* HMPV + HPIV-1 + HPIV-3 + *S. pneumoniae* + *M. pneumoniae* HMPV + HPIV-1 + HRV A/B/C HMPV + HPIV-1 + EV + *S. pneumoniae* + *M. pneumoniae* HMPV + HBoV 1/2/3/4 + HAdV + *S. pneumoniae*	1 (0.2) 1 (0.2) 1 (0.2) 1 (0.2) 1 (0.2)
EV + HAdV + HCoV OC43 + *H. influenzae*	1 (0.2)
EV + HRV A/B/C + *H. influenzae* EV + HPIV-2 + HRV A/B/C + *S. pneumoniae* EV + HPIV-3 + HRV A/B/C + *S. pneumoniae* + *H. influenzae* EV + HMPV + *S. pneumoniae* EV + HPIV-1 + HRV A/B/C + *H. influenzae*	1 (0.2) 1 (0.2) 1 (0.2) 1 (0.2)1 (0.2)
HPIV-4 + HRV A/B/C + *S. pneumoniae* + *H. influenzae*	1 (0.2)
HPIV-4 + HBoV 1/2/3/4 + HRV + *S. pneumoniae* HPIV-4 + *M. pneumoniae* + *H. influenzae* HPIV-4 + HBoV 1/2/3/4 + *H. influenzae* HPIV-4 + HRV A/B/C + *S. pneumoniae*	1 (0.2) 1 (0.2) 1 (0.2) 1 (0.2)
HPIV-2 + *S. pneumoniae* + *H. influenzae* HCoV NL63 + HCoV 229E	1 (0.2) 1 (0.2)
HCoV NL63 + HPMV + *H. influenzae*	1 (0.2)
HCoV NL63 + HAdV + *S. pneumoniae* + *H. influenzae*	1 (0.2)
HCoV NL63 + *S. pneumoniae* + *H. influenzae*	1 (0.2)
HCoV NL63 + HRV A/B/C + *H. influenzae* HCoV NL63 + HRV A/B/C + *S. pneumoniae* + *H. influenzae* HCoV NL63 + HAdV + *S. pneumoniae* + *H. influenzae* + *M. pnemoniae* HCoV NL63 + HBoV 1/2/3/4 + *S. pneumonie* + *H. influenzae* HPIV-3 + EV + *H. influenzae* HPIV-3 + HBoV 1/2/3/4 + EV *B. parapertussis* + *H. influenzae* + *S. pneumonie*	1 (0.2) 1 (0.2) 1 (0.2) 1 (0.2) 1 (0.2) 1 (0.2) 1 (0.2)

HRV: Human rhinovirus; HAdV: Human adenovirus; HBoV: Human bocavirus; HMPV: Human metapneumovirus; HPIV: Human parainfluenza virus; EV: Enterovirus; RSV: Respiratory syncytial virus; HCoV: Human coronaviruses; FLU A/B: Influenza A/B.

**Table A2:** Distribution of viral pathogens by gender.

Viruses	Boys (%) *n* = 458	Girls (%) *n* = 345	Total (%) *n* = 803	*p* Value
Viral monoinfections Viral coinfections Mixed coinfections Bacterial monoinfections Bacterial coinfections Positive tests	123 (26.9)	72 (20.9)	195 (24.3)	0.442
	34 (7.4) 192 (41.9) 41 (9) 14 (3.1)	21 (6.1) 165 (47.8) 32 (9.3) 9 (2.6)	55 (6.9) 357 (44.5) 73 (9.1) 23 (2.9)	
	404 (88.2)	299 (86.7)	703 (87.6)	
HRV A/B/C	178 (38.9)	131 (38)	309 (38.5)	0.797
HAdV	60 (13.1)	50 (14.5)	110 (13.7)	0.570
HBoV 1/2/3/4	48 (10.5)	43 (12.5)	91 (11.3)	0.380
HMPV	30 (6.6)	22 (6.4)	52 (6.5)	0.921
HPIV-1	7 (1.5)	6 (1.7)	13 (1.6)	0.815
HPIV-2	2 (0.4)	2 (0.6)	4 (0.5)	1.000
HPIV-3	29 (6.3)	20 (5.8)	49 (6.1)	0.754
HPIV-4	14 (3.1)	8 (2.3)	22 (2.7)	0.526
EV	23 (5)	16 (4.6)	39 (4.9)	0.802
RSV	23 (5)	20 (5.8)	43 (5.4)	0.629
HCoV NL63	14 (3.1)	10 (2.9)	24 (3)	0.896
HCoV OC43	14 (3.1)	10 (2.9)	24 (3)	0.896
HCoV 229E	2 (0.4)	0 (0)	2 (0.3)	0.509
SARS-CoV-2	10 (2.2)	7 (2)	17 (2.1)	1.000
FLUA	4 (0.9)	3 (0.9)	7 (0.9)	1.000
FLUB	1 (0.2)	1 (0.3)	2 (0.3)	1.000

HRV: Human rhinovirus; HAdV: Human adenovirus; HBoV: Human bocavirus; HMPV: Human metapneumovirus; HPIV: Human parainfluenza virus; EV: Enterovirus; RSV: Respiratory syncytial virus; HCoV: Human coronaviruses; FLU A/B: Influenza A/B.

**Table A3:** Correlation of pathogens with respiratory tract infections.

Viruses	URTI *n* (%) *n* = 108	LRTI *n* (%) *n* = 694	*p* Value
HRV A/B/C	30 (27.8)	279 (40.2)	0.014 *
HAdV	23 (21.3)	87 (12.5)	0.014 *
HBoV 1/2/3/4	6 (5.6)	84 (12.1)	0.045 *
HMPV	4 (3.7)	48 (6.9)	0.207
HPIV-1	1 (0.9)	12 (1.7)	1.000
HPIV-2	0	4 (0.6)	1.000
HPIV-3	3 (2.8)	46 (6.6)	0.120
HPIV-4	4 (3.7)	18 (2.6)	0.522
EV	6 (5.6)	33 (4.8)	0.719
RSV	0	43 (6.2)	0.008 *
HCoV NL63	2 (1.9)	22 (3.2)	0.760
HCoV OC43	4 (3.7)	20 (2.9)	0.552
HCoV 229E	1 (0.9)	1 (0.1)	0.251
SARS-CoV-2	7 (6.5)	10 (1.4)	0.004 *
FLU A	1 (0.9)	6 (0.9)	1.000
FLU B	0	2 (0.3)	1.000
*S. pneumoniae*	26 (24.1)	285 (41.1)	0.001 *
*H. influenzae*	28 (25.9)	252 (21.8)	0.035 *
*B. parapertussis*	0	12 (1.7)	0.386
*M. pneumoniae*	3 (2.8)	5 (0.7)	0.080

* *p < 0.05*. HRV: Human rhinovirus; HAdV: Human adenovirus; HBoV: Human bocavirus; HMPV: Human metapneu-movirus; HPIV: Human parainfluenza virus; EV: Enterovirus; RSV: Respiratory syncytial virus; HCoV: Human coronaviruses; FLU A/B: Influenza A/B.

**Table A4:** Correlation of respiratoy pathogens with PICU admission and O2 therapy.

Viruses	PICU *n* (%)	*p* Value	O2 Therapy *n* (%)	*p* Value
HRV A/B/C (n = 309)		0.164		0.583
Monoinfection	17 (5.5)		4 (1.3)	
Viral coinfection	9 (2.9)		2 (0.7)	
Mixed coinfection	50 (16.2)		9 (2.9)	
HAdV (n = 110)		0.174		1.000
Monoinfection	1 (0.9)		0	
Viral coinfection	2 (1.8)		0	
Mixed coinfection	13 (11.8)		3 (2.7)	
HBoV 1/2/3/4 (n = 91)		0.394		0.654
Monoinfection	3 (3.3)		1 (1.1)	
Viral coinfection	4 (4.4)		1 (1.1)	
Mixed coinfection	16 (17.6)		2 (2.2)	
HMPV (n = 52)		0.787		1.000
Monoinfection	2 (3.9)		0	
Viral coinfection	1 (1.9)		0	
Mixed coinfection	9 (17.3)		1 (1.9)	
HPIV-1 (n = 13)		0.326		0.002 *
Monoinfection	1 (7.7)		2 (15.4)	
Viral coinfection	1 (7.7)		0	
Mixed coinfection	2 (15.4)		1 (7.7)	
HPIV-2 (n = 4)		0.612		1.000
Monoinfection	0		0	
Mixed coinfection	1 (25)		0	
HPIV-3 (n = 49)		0.757		1.000
Monoinfection	3 (6.1)		0	
Viral coinfection	1 (2)		0	
Mixed coinfection	3 (6.1)		0	
HPIV-4 (n = 22)		0.862		1.000
Monoinfection	2 (9.1)		0	
Viral coinfection	0		0	
Mixed coinfection	3 (13.6)		0	
EV (n = 39)		0.601		0.124
Monoinfection	0		1 (2.6)	
Viral coinfection	0		0	
Mixed coinfection	6 (15.4)		2 (5.1)	
RSV (n = 43)		0.773		0.102
Monoinfection	3 (7)		2 (4.7)	
Viral coinfection	3 (7)		1 (2.3)	
Mixed coinfection	3 (7)		1 (2.3)	
HCoV NL63 (n = 24)		0.908		1.000
Monoinfection	0		0	
Viral coinfection	0		0	
Mixed coinfection	3 (12.5)		0	
HCoV OC43 (n = 24)		0.952		1.000
Monoinfection	1 (4.2)		0	
Viral coinfection	0		0	
Mixed coinfection	2 (8.3)		0	
HCoV 229E (n = 2)		1.000		1.000
Viral coinfection	0		0	
Mixed coinfection	0		0	
SARS-CoV-2 (n = 17)		0.017 *		1.000
Monoinfection	3 (17.7)		0	
Viral coinfection	2 (11.8)		0	
Mixed coinfection	3 (17.7)		0	
FLU A (n = 7)		0.282		1.000
Monoinfection	0		0	
Viral coinfection	1		0	
Mixed coinfection	0		0	
FLU B (n = 2)		0.377		1.000
Monoinfection	1 (14.3)		0	
Viral coinfection	0		0	
*S. pneumoniae* (n = 311)		0.472		0.636
Monobacterial infection	10 (3.2)		1 (0.3)	
Bacterial coinfection	5 (1.6)		1 (0.3)	
Mixed coinfection	59 (19)		7 (2.3)	
*H. influenzae* (n = 278)		0.337		0.128
Monobacterial infection	2 (0.7)		0	
Bacterial coinfection	5 (1.8)		1 (0.4)	
Mixed coinfection	49 (17.6)		4 (1.4)	
*B. parapertussis* (n = 12)		1.000		0.379
Monobacterial infection	0		0	
Bacterial coinfection	1 (8.3)		0	
Mixed coinfection	1 (8.3)		1 (8.3)	
*M. pneumoniae* (n = 8)				1.000
Mixed coinfection	1 (12.5)	1.000	0	

* *p* < 0.05. HRV: Human rhinovirus; HAdV: Human adenovirus; HBoV: Human bocavirus; HMPV: Human metapneu-movirus; HPIV: Human parainfluenza virus; EV: Enterovirus; RSV: Respiratory syncytial virus; HCoV: Human coronaviruses; FLU A/B: Influenza A/B.
